# In-Motion Alignment with MEMS-IMU Using Multilocal Linearization Detection

**DOI:** 10.3390/s25092645

**Published:** 2025-04-22

**Authors:** Yulu Zhong, Xiyuan Chen, Ning Gao, Zhiyuan Jiao

**Affiliations:** 1 School of Instrument Science and Engineering, Southeast University, Nanjing 210018, China; yulu_zhong@seu.edu.cn (Y.Z.); ninggaoseu@seu.edu.cn (N.G.); jiaozhiyuan@seu.edu.cn (Z.J.); 2 The Key Laboratory of Micro-Inertial Instrument and Advanced Navigation Technology of Ministry of Education, Southeast University, Nanjing 210018, China

**Keywords:** initial alignment, in-motion alignment, generalized Schweppe likelihood ratio, multilocal linearization, extended Kalman filter, quasi-uniform quaternion generation method

## Abstract

In-motion alignment is a critical step in obtaining the initial state of an integrated navigation system. This article considers the in-motion initial alignment problem using the multilocal linearization detection method. In contrast to the OBA-based method, which fully relies on satellite signals to estimate the initial state of the Kalman filter, the proposed method utilizes the designed quasi-uniform quaternion generation method to estimate several possible initial states. Then, the proposed method selects the most probable result based on the generalized Schweppe likelihood ratios among multiple hypotheses. The experiment result of the proposed method demonstrates the advantage of estimation performance within poor-quality measurement conditions for the long-duration coarse alignment using MEMS-IMU compared with the OBA-based method. The proposed method has potential applications in alignment tasks for low-cost, small-scale vehicle navigation systems.

## 1. Introduction

At the startup of an integrated navigation system, the orientation of its body frame relative to the reference navigation frame is unknown, preventing it from immediately entering the navigation state. Therefore, it is essential to determine the spatial orientation of the body frame with respect to the navigation frame [1]. From another perspective, the essence of integrated navigation is solving nonlinear differential equations, so accurately determining the initial state of the navigation system contributes to the convergence of the solution. Typically, three aspects compose the conventional initial alignment process: (1) the vehicle motion state, (2) the attitude determination method, and (3) the state estimation method. Wahba’s problem plays a central role in attitude determination, in which the TRIAD algorithm uses two measurement vectors to determine a coarse attitude, and the widely used quaternion estimator (QUEST) algorithm uses more measurement vectors [2]. A coarse attitude is rapidly derived from a double/multi-measurement vector when the vehicle is stationary or quasi-stationary. However, the large misalignment attitude of coarse alignment means that the next fine alignment procedure cannot quickly converge to an accurate result. Specifically, the converging iteration takes so much time that the conventional state estimator is hardly used in such in-motion cases, e.g., drifting watercraft alignment, missile launching, in-flight aircraft alignment, etc. As for in-flight motion, the optimization-based alignment (OBA) aided by a Global Positioning System (GPS) [3] utilizes the *q*-method algorithm [2] to recursively acquire a coarse initial attitude. Although the IFA-VIF/PIF proposed by Wu can determine a coarse attitude at a motion state, the determination result has great sensitivity to the sensor noise, especially the bias noise. Soon, a patch version that can jointly estimate attitude and other associated initialization parameters is successfully used in high-quality INS and GPS equipment initial alignment [4]. Meanwhile, a dynamic OBA method with sliding windows and a quaternion estimator is proposed to estimate gyroscope biases coupled with attitude for low-grade SINS [5,6]. Then, a state estimator is immediately switched to accomplish fine alignment after the coarse alignment runs for some time. Based on the dynamic OBA method, several modified coarse alignment methods have aimed at computational efficiency and accuracy over the last five years [7,8].

Normally, the state estimator consists of attitude representation and estimation methods. The four-component unit quaternion relies on its global nonsingularity as a dominant attitude parameterization. The challenge with using the extended Kalman filter for attitude estimation with quaternions is that quaternions must always be normalized. This requirement means that the Gaussian distribution cannot be guaranteed for quaternions that reside on the unit sphere SO(3). Interestingly, a three-component vector can be used to represent the attitude error by neglecting higher-order terms. Therefore, the attitude error kinematics based on multiplicative error quaternion is derived to propagate EKF covariance which is called the MEKF method [9]. In return, the MEKF can be applied in the quaternion-based nonlinear error model [10,11] and the basic model for in-flight alignment [12], respectively. To improve the performance of its nonlinear estimator, a sigma-point Kalman filter uses an unscented transformation to approximate the state distribution as a Gaussian distribution in cases of large initial attitude errors [13]. Meanwhile, a generalized Rodrigues parameter is used to represent the multiplicative error quaternion for attitude estimation in the UnScented Quaternion Estimator (USQUE) [14] method that can expand to apply to a GPS/INS loose couple based on the basic model [12,15].

Although fine alignment mitigates the nonlinearity induced by large misalignment angles, its convergence time remains influenced by the accuracy of coarse alignment [16,17,18]. However, no specified criterion defines the optimal timing for transitioning from coarse alignment to fine alignment. The aforementioned coarse alignment methods require a substantial amount of continuous and reliable velocity and position data to ensure alignment accuracy. However, in complex environments such as tunnels and areas with trees, satellite signals are often intermittent, which significantly disrupts the accuracy of coarse alignment results. Therefore, this study considers employing a multilocal linearization detection approach to address this problem. The study uses a multitude of potential initial attitude estimates and generalized Schweppe and Gaussian likelihood ratios [19,20] for selecting the most likely of those initial estimates [21,22]. Hence, the proposed method utilizes a nonlinear state estimator to account for sensor uncertainties, achieving superior estimation accuracy compared to traditional methods. Specifically, this study has the following primary contributions:

1. To the best of the authors’ knowledge, the maximum likelihood of the generalized Schweppe likelihood ratio method is applied for the first time to replace the coarse alignment process. The proposed method only requires satellite signals at two-time instants to make the initial estimates.
2. The proposed quasi-uniform quaternion method in this study significantly reduces the number of initial estimates required.
3. The vehicle experiment is designed and implemented to evaluate the proposed method.

The remainder of this paper is organized as follows. The attitude alignment process and the problem of the existing method in initial alignment are stated in Section 2. Section 3 shows the key points of the proposed method. Section 4 evaluates the proposal method through experiments with GPS/IMU data from the outfield test. Section 5 discusses the performance of the proposed method. Finally, Section 6 summarizes the contribution of this work.

## 2. Mathematical Models and Problem Statement

### 2.1. Mathematical Models

The attitude quaternion q is denoted as ${\left[\begin{array}{cc} q_{0} & \varrho \end{array}\right]}^{T}$, vehicle velocity is denoted as $v^{n}$, and the vehicle position is denoted as $p^{n}$. The inertial frame is *i*; the body frame is *b*; the latitude, longitude, and height are denoted as *L*, λ, and *h*, respectively; and the navigation frame is denoted as *n*. Then, the basic model equations are given by the following equations: [11,13,23]

(1a) $$\begin{array}{c} {\dot{q}}_{b}^{n}=\frac{1}{2}q_{b}^{n}⊗\omega_{nb}^{b}\\ \omega_{nb}^{b}=\omega_{ib}^{b}-\omega_{in}^{b} \end{array}$$

(1b) $$\begin{array}{c} {\dot{v}}^{n}=A\left(q_{b}^{n}\right)f_{sf}^{b}-\left(2\omega_{ie}^{n}+\omega_{en}^{n}\right)\times v^{n}+g^{n}\\ A\left(q\right)=(q_{0}^{2}-{∥\varrho ∥}^{2})I_{3}-2q_{0}\left[\varrho \times \right]+2\varrho \varrho^{T} \end{array}$$

(1c) $$\begin{array}{c} {\dot{p}}^{n}=R_{c}v^{n}\\ R_{c}=\left[\begin{array}{ccc} 0 & \frac{1}{R_{M}+h} & 0\\ \frac{\sec L}{R_{N}+h} & 0 & 0\\ 0 & 0 & 1 \end{array}\right] \end{array}$$

where $R_{M}$ and $R_{N}$ are the principal radii of curvature along the meridional section and the prime section, respectively. $\omega_{nb}^{b}$ is the angular rate of *n* frame relative to *b* frame in *n* frame. $\omega_{ib}^{b}$ is the body angular rate. $\omega_{in}^{b}$ is the angular rate of *i* frame relative to *n* frame in *b* frame. The navigation state vector is defined as

(2) $$x={\left[\begin{array}{ccccc} q_{b}^{nT} & v^{nT} & p^{nT} & \epsilon_{g}^{T} & \nabla_{a}^{T} \end{array}\right]}^{T}$$

where $\epsilon_{g}$ and $\nabla_{a}$ are the gyroscope and accelerometer bias, respectively. It is reasonable for a small initial attitude error that using rotation vector α form to represent the multiplicative error quaternion δq, i.e., $\delta q=[\cos(∥\alpha ∥),\sin\left(\alpha \right)\alpha /{∥\alpha ∥]}^{T}$. The error kinematics of estimation follows:

(3) $$\dot{\hat{\alpha }}=-\left[{\hat{\omega }}_{in}^{n}\times \right]\hat{\alpha }-\delta \omega_{in}^{n}+A\left({\hat{q}}_{b}^{n}\right)\delta \omega_{ib}^{b}$$

where $\delta \omega_{ib}^{b}=\omega_{ib}^{b}-{\tilde{\omega }}_{ib}^{b}$, and the $\delta \omega_{in}^{n}$ can be written as

(4) $$\begin{array}{cc} \delta \omega_{in}^{n} & =\delta \omega_{ie}^{n}+\delta \omega_{en}^{n}\\ \; & =\left(M_{1}+M_{3}\right)\delta p^{n}+M_{2}\delta v^{n}\\ M_{1} & =\left[\begin{array}{ccc} 0 & 0 & 0\\ -\omega_{ie}\sin L & 0 & 0\\ \omega_{ie}\cos L & 0 & 0 \end{array}\right]\\ M_{2} & =\left[\begin{array}{ccc} 0 & -\frac{1}{R_{M}+h} & 0\\ \frac{1}{R_{N}+h} & 0 & 0\\ \frac{\tan L}{R_{N}+h} & 0 & 0 \end{array}\right]\\ M_{3} & =\left[\begin{array}{ccc} 0 & 0 & \frac{v_{N}^{n}}{{\left(R_{M}+h\right)}^{2}}\\ 0 & 0 & -\frac{v_{E}^{n}}{{\left(R_{N}+h\right)}^{2}}\\ \frac{v_{E}^{n}\sec^{2}L}{R_{N}+h} & 0 & -\frac{v_{E}^{n}\tan L}{{\left(R_{N}+h\right)}^{2}} \end{array}\right] \end{array}$$

where $\delta p^{n}=p^{n}-{\hat{p}}^{n}$, and $\delta v^{n}=v^{n}-{\hat{v}}^{n}$. Then (1b) can be rewritten as

(5a) $${\dot{\hat{v}}}^{n}=A\left({\hat{q}}_{b}^{n}\right)\left(I_{3}+\left[\hat{\alpha }\times \right]\right){\hat{f}}_{sf}^{b}-\left(2{\hat{\omega }}_{ie}^{n}+{\hat{\omega }}_{en}^{n}\right)\times {\hat{v}}^{n}+{\hat{g}}^{n}$$

(5b) $${\dot{\hat{p}}}^{n}={\hat{R}}_{c}{\hat{v}}^{n}$$

where the circumflex denotes the estimation of the corresponding variable.

### 2.2. Problem Statement

**Figure 1:**
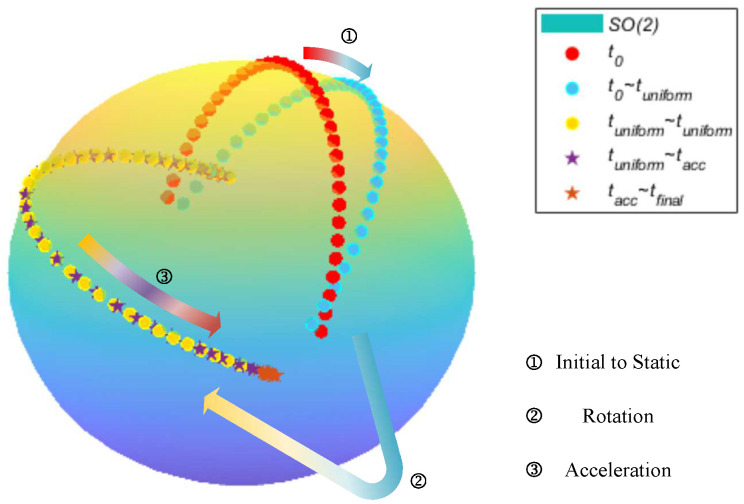
The attitude initial alignment process in SO(3).

From the specific force Equation (5a), it is evident that the navigation model is a nonlinear model, with its nonlinearity increasing as the initial attitude error grows. Therefore, to obtain a convergent solution for the solution of the nonlinear navigation equations, it is essential to acquire a sufficiently accurate initial state. The OBA-based methods establish vectors based on the specific force Equation (5a) and employ the *q*-method to solve the initial attitude-determined problem. The attitude-determined problem of the initial alignment is depicted in the SO(3) as Figure 1. The vehicle motion is divided into stationary, rotational, and accelerated phases. At the initial time, due to the lack of attitude angle information in one dimension, the state is initialized as a uniform distribution over SO(3). In the first and second phases, the distribution will remain unchanged owing to the absence of new information. In the third phase, the state begins to gradually converge as the accelerated motion enhances the observability of the unknown attitude angles.

According to previous research, this is a nonlinear problem in the context of filtering. The most common solution, as mentioned in the introduction, is to employ the aforementioned coarse alignment methods to obtain an initial estimate with minimal error, thereby avoiding issues such as nonlinear filter divergence and prolonged convergence times. However, in complex environments, intermittent satellite signals can easily degrade the accuracy of coarse alignment, resulting in a long convergence time for fine alignment. Additionally, the switching time from coarse alignment to fine alignment is ambiguous. Both insufficient and excessive coarse alignment durations can also degrade the accuracy of the results.

## 3. Multilocal Linearization and Maximum Likelihood Estiamtion

### 3.1. The Proposed Method Framework

**Figure 2:**
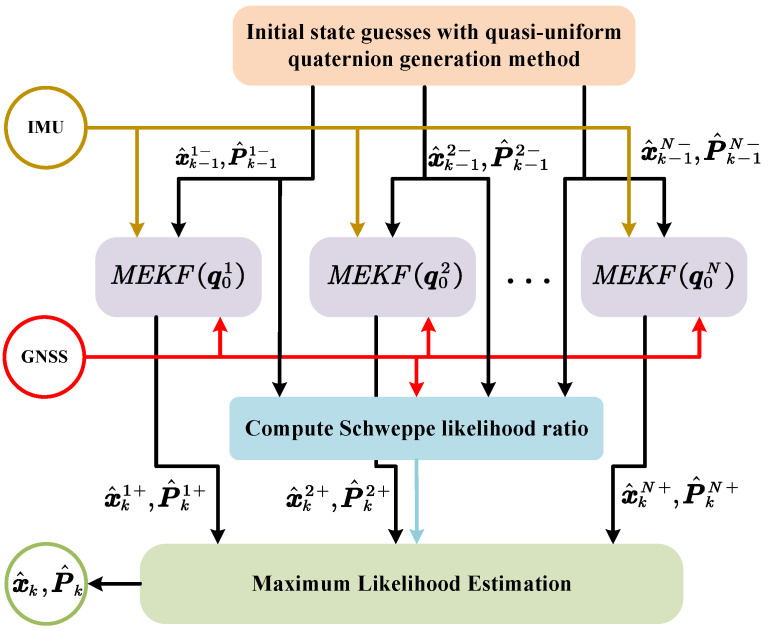
The proposed method framework.

The designed algorithm framework is depicted in Figure 2. Firstly, the filters are initialized by the proposed quasi-uniform quaternion generation method. Then, the Schweppe likelihood ratio between filters is computed based on the innovation and estimated distribution. Finally, the maximum likelihood estimation is used to select the most probable initial estimate.

### 3.2. Initial Quasi-Uniform Quaternion Generation Method for Multilocal Linearization

**Figure 3:**
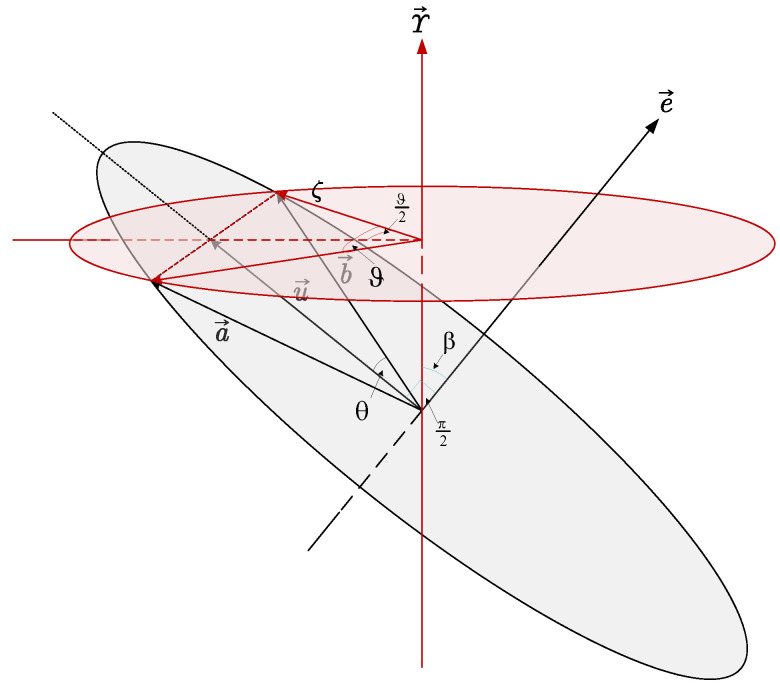
Quasi-uniform quaternion generation method.

A straightforward mathematical meaning method is proposed to generate the initial state for each model. Denoting the first observation vectors as $\vec{a}$ and $\vec{b}$, the relationship of this pair of the vector is $\vec{a}=A\left(q\right)\vec{b}$. It can be said with certainty that the rotation quaternion is rarelyderived from only one pair of vectors. However, it can be asserted that the rotation quaternion must exist in the plane constructed by $\vec{u}$ and $\vec{e}$, as shown in Figure 3.

The cross product $\vec{e}$, the vectorial sum $\vec{u}$, and the vector angle θ of the pair vectors are given by (6)

(6) $$\begin{array}{c} \vec{u}=\left(\vec{a}+\vec{b}\right)/2\;\vec{e}=\vec{a}\times \vec{b}/∥\vec{a}\times \vec{b}∥\\ \theta =\frac{1}{2}\arccos({\vec{b}}^{T}\vec{a}/∥\vec{b}∥∥\vec{a}∥) \end{array}$$

The rotation axis is given by (7)

(7) $$\vec{\gamma }=\vec{e}\cos\beta +\frac{\vec{u}}{∥\vec{u}∥}\sin\left(\beta \right)$$

The quaternion can be derived by (8)

(8) $$\begin{array}{c} q_{0}=\cos\frac{\vartheta }{2}=\cos\theta \cos\beta /\zeta \\ \varrho =\sin\frac{\vartheta }{2}\vec{\gamma }=\sin\theta \vec{\gamma }/\zeta \\ \zeta =\sqrt{\sin^{2}\theta +\cos^{2}\theta \cos^{2}\beta } \end{array}$$

The uniform rotation axis quaternion can be achieved by rotating β from 0 to π. Moreover, the rotation angle of quaternion ϑ/2 also needs to be assumed to have a uniform distribution between θ∼π−θ, due to the redundancy of quaternion. Then, β can be set as (9)

(9) $$\beta =\arccos(\frac{\tan\theta }{\tan\frac{\vartheta }{2}})$$

**Figure 4:**
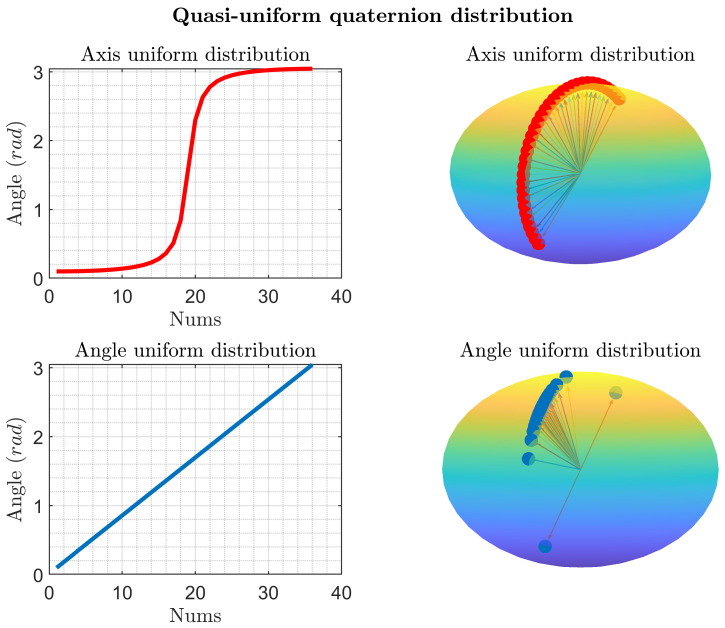
Quasi-uniform quaternion distribution.

The quasi-uniform quaternion estimates can be obtained, as shown in Figure 4

The observation vector pair can be constructed using only the GPS information from two consecutive observations based on the OBA method [3]. Note that the observation vector pair is constructed using the OBA method because OBA is more robust during vehicle motion, when the vehicle is stationary, the vector pair can be constructed using the gravitational acceleration and the accelerometer output. The fundamental difference between the proposed method and the OBA method lies in the fact that the OBA method uses an attitude determination approach based on solving Wahba’s problem, while the proposed method directly estimates the navigation state using a nonlinear filtering approach.

### 3.3. Generalized Schweppe Likelihood and Maximum Likelihood Estiamtion

The navigation system model [23] can usually be expressed as (10)

(10) $$\begin{array}{cc} x_{k} & =f\left(x_{k-1}\right)+w_{k-1}\\ y_{k} & =h\left(x_{k}\right)+v_{k} \end{array}$$

where $x_{k}\in R^{n}$ is the state and $y_{k}\in R^{p}$ is the measurement in the step *k*; the nonlinear functions f(·) and h(·) are the dynamic and measurement model, respectively. The $w_{k}$ is the process noise that conforms to the Gaussian white noise with variance $Q_{k-1}$. Correspondingly, the $v_{k}$ is the measurement of Gaussian white noise with variance $R_{k}$. Let *m* denote the number of the initial estimates. As is well known, the Kalman filter provides an estimate of the measurement distribution. With *m* different initial hypotheses, *m* different estimates of the measurement distribution can be obtained. Then, at time *t*, the likelihood probability of the measurement distribution estimate for one of the *m* hypothesized measurement distributions can be expressed as (11).

(11) $$p\left(y_{1:t}|H_{i}\right)=\prod_{k=1}^{t}p\left(\nu_{k,i}\right)=\prod_{k=1}^{t}{\left|P_{\nu_{k}\nu_{k},i}\right|}^{\frac{1}{2}}\exp\left(-\frac{1}{2}\nu_{k,i}^{T}P_{\nu_{k}\nu_{k},i}^{-1}\nu_{k,i}\right)$$

where $H_{i}$ denotes the *i* initial hypotheses; $\nu_{k}$ is the time *k* innovation between the measurement and prediction; and $P_{\nu_{k}\nu_{k}}$ is the covariance of $\nu_{k}$, which can be expressed as (12) with Kalman theory.

(12) $$\begin{array}{cc} \nu_{k,i} & =y_{k}-x_{k,i}^{-}\\ P_{\nu_{k}\nu_{k},i} & =H_{k,i}^{T}P_{xx,i}^{-}H_{k,i}+R \end{array}$$

where $x_{k,i}^{-}$ and $P_{xx,i}^{-}$ are the predicted state and covariance from the time update of Kalman filter. Then, the likelihood ratio between any two hypotheses can be written as (13)

(13) $$\lambda_{ij}=\log\frac{p(y_{1:t}|H_{i})}{p(y_{1:t}|H_{j})}=\sum_{k=1}^{t}-\frac{1}{2}\nu_{k,i}^{T}P_{\nu_{k}\nu_{k},i}^{-1}\nu_{k,i}+\frac{1}{2}\nu_{k,j}^{T}P_{\nu_{k}\nu_{k},j}^{-1}\nu_{k,j}+\frac{1}{2}\left(\right|P_{\nu_{k}\nu_{k},i}\left|-\right|P_{\nu_{k}\nu_{k},j}\left|\right)$$

Then, the maximum likelihood is used to select the state corresponding to the maximum likelihood ratio, which can be expressed as (14)

(14) $${\hat{x}}_{t}=\arg\max_{x^{+}}\left(\lambda_{ij}\right)\;1\leq i\leq m,1\leq j\leq m$$

where $x^{+}$ denotes the posterior state of the Kalman filter.

## 4. Experiments and Discussion

### 4.1. Experiment Setup

The data and the proposed method of the experiment can be obtained at https://github.com/zhongluu/CoarseAlignment.git (accessed on 28 February 2025). The parameters of the experiment sensors are shown in Table 1.

To evaluate the performance of the proposed method, the comparison methods consist of four approaches: the USQUE with large misalignment angle $60^{{}^{\circ}}$ (denote as USQUE) [14], the USQUE [14] with coarse alignment (OBA) [3], the coarse alignment with attitude determination method (denote as Wu OBA) [3], and the coarse alignment with dynamic OBA and Kalman filter (denote as Huang IMCA) [8]. The filter parameters of these employed state estimators are set as $P_{0}=diag(10^{{}^{\circ}}\;I_{1\times 3},\;0.15\;m/s\;I_{1\times 3},\;3\;m/s\;I_{1\times 3},\;250^{{}^{\circ}}/h\;I_{1\times 3}$, $750\;\mu g\;I_{1\times 3})$, $Q=diag(0.15^{{}^{\circ}}/\sqrt{h},\;0.07\;m/s/\sqrt{h})$, and R=diag(0.15m/s,3m) for experiential tests, respectively.

### 4.2. Vehicle Experiment

**Figure 5:**
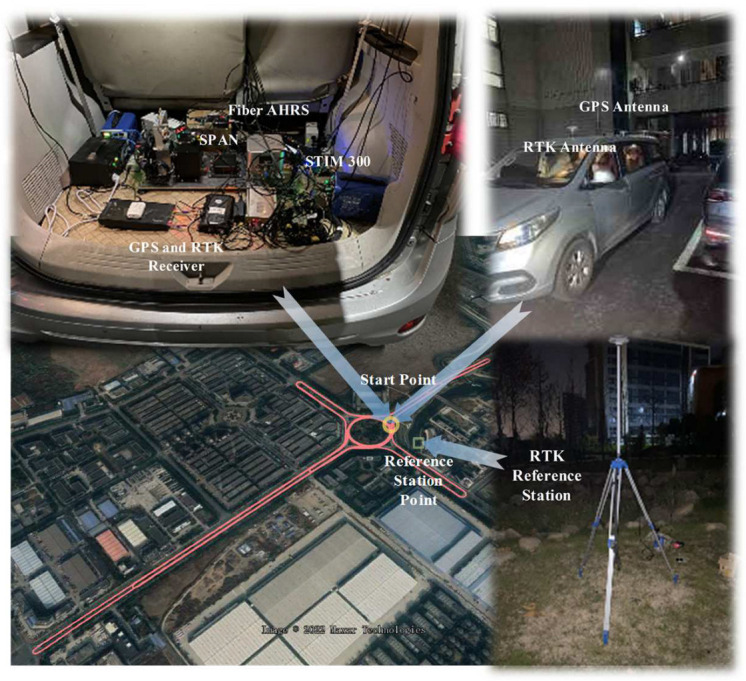
The experiment environment of vehicle experiment.

The experiment equipment, trajectory, and environment are plotted in Figure 5. The SPAN integrated navigation system, which combines a MEMS IMU and GPS, along with a high-performance AHRS integrated navigation system that combines a fiber optic gyroscope and RTK, are jointly installed on a vehicle-mounted platform. The motion information of the vehicle is simultaneously collected in real time by both systems. The specified instrument characteristics are listed in Table 1.

**Figure 6:**
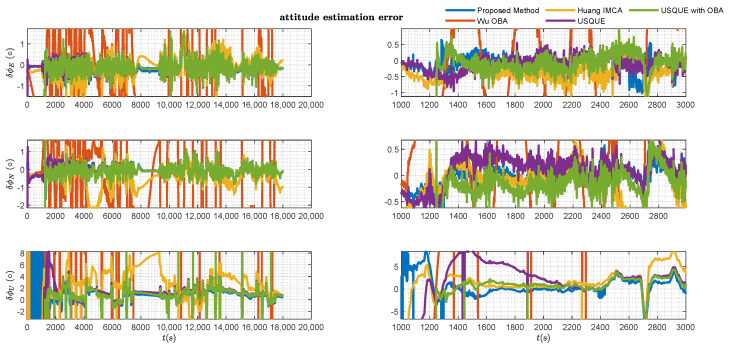
Attitude estimation error of various filters.

The left side of Figure 6 plots an overview of the attitude estimation error results (with respect to pitch, roll, and yaw) for different methods throughout the entire alignment period. In the initial few minutes, as the vehicle remains stationary, the observability of the yaw is weak, making alignment impossible with a MEMS-IMU. Consequently, none of the methods can provide an accurate yaw estimate. The Wu OBA method, lacking bias estimation for the inertial sensors, exhibits corresponding drift in pitch and roll. The proposed method, due to the small differences in likelihood ratios among multiple hypotheses, results in the frequent switching of the maximum likelihood hypothesis, leading to the yaw estimation error result in the initial period of the figure. Additionally, from the left half of the figure, it can be observed that for long-duration alignment, these OBA methods not only fail to improve alignment accuracy but also lead to its degradation. Meanwhile, the right side of Figure 6 plots the detailed attitude estimation error result of various methods after the vehicle started. It can be seen from Figure 6 that the estimations of these coarse alignment methods with the OBA method are inferior to other methods. However, the USQUE is at risk of divergence when the misalignment angle is too large. The transition from coarse alignment to fine alignment in USQUE with the OBA method is manually determined. From Figure 6, it can also be seen that, regardless of whether in the initial time period or over the entire duration, the estimation performance of the proposed method is comparable to that of the USQUE with OBA method, which employs manual coarse alignment switching.

**Figure 7:**
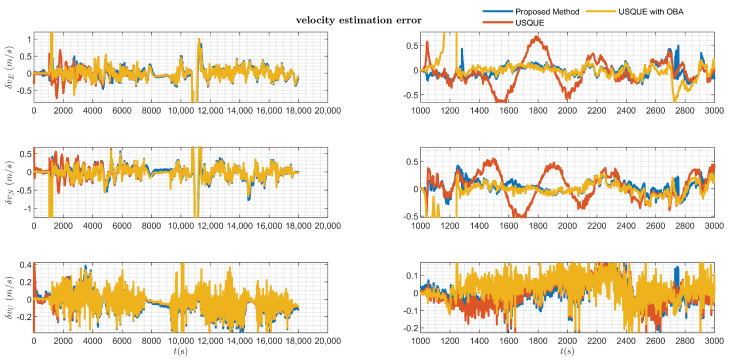
Velocity estimation error of various filters.

**Figure 8:**
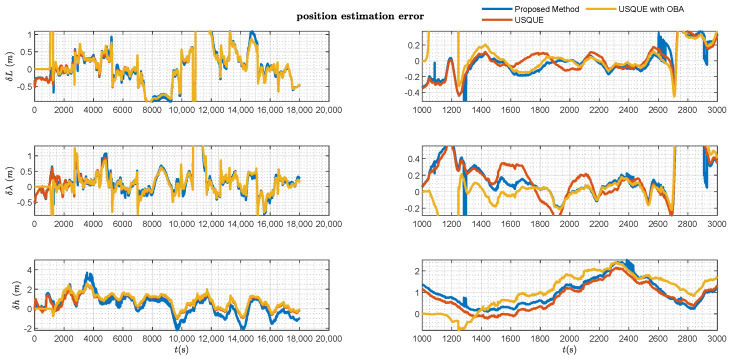
Position estimation error of various filters.

**Figure 9:**
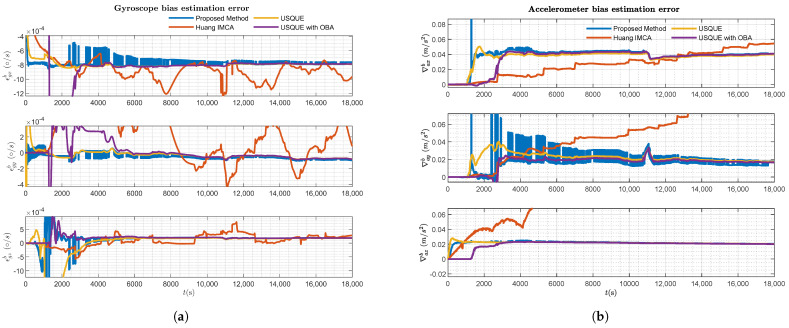
Sensors bias estimation result of various filters: (**a**) Gyroscope bias estimation. (**b**) Accelerometer bias estimation.

The estimation error results of the various filters are presented in Figure 6, Figure 7, Figure 8 and Figure 9.

**Figure 10:**
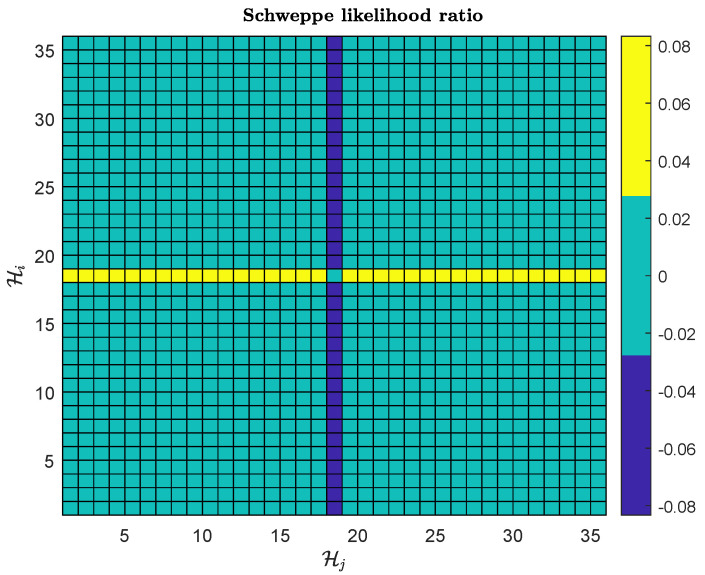
Generalized Schweppe likelihood ratio.

Figure 7 and Figure 8, respectively, present the velocity and position estimation errors in northern and eastern directions for various methods. Consistent with Figure 6, the left half of Figure 7 and Figure 8 present an overview of the estimation errors for different methods throughout the entire alignment period, while the right halves provide a detailed view of the errors during a period after the vehicle motion. The OBA-based coarse alignment method is absent in velocity and position estimation due to its complete reliance on satellite information for alignment. Additionally, from Figure 7 and Figure 8, it can be observed that the estimation performance of the USQUE with a large misalignment angle method is clearly inferior to that of the proposed method within the initial convergence time of USQUE. The proposed method also demonstrates a performance similar to that of the USQUE method with the OBA approach based on manual switching. Meanwhile, due to the large misalignment, the USQUE method exhibits fluctuations in velocity and position errors during the initial period in the right half of Figure 7 and Figure 8. Figure 9 shows the bias estimation results of the gyroscope and accelerometer. Correspondingly, the OBA-based coarse alignment method has demonstrated inferior estimation performance overall, consistent with the previous estimations. Interestingly, the estimation results of the proposed method show a less smooth curve with a sawtooth-like pattern. This indicates that as the alignment time progresses, the likelihood ratios among multiple hypotheses continuously change, leading to shifts in the hypothesis with the highest likelihood ratio, which, in turn, results in a sawtooth-like waveform. However, the estimation curve of the proposed method gradually converges with the increasing alignment time. Finally, the likelihood ratio of the proposed method at the lowest alignment time is plotted in Figure 10. The RMSE errors of various methods at the time of alignment are listed in Table 2. From Table 2, it can be observed that the estimation error of the proposed method shows an advantage over the OBA-based methods and is comparable to the results of the manual switching alignment method. Moreover, since the OBA-based methods rely entirely on the velocity and position information provided by GPS, the corresponding error terms are missing. Furthermore, since the proposed method employs MEKF, whereas USQUE is a nonlinear filtering method based on sigma point propagation, the proposed method presents slightly inferior velocity and position estimation compared to the USQUE method. However, the primary concern is the estimation of the vehicle attitude for initial alignment.

## 5. Discussion

According to the experiment results, the performance of the OBA-based method is inferior to that of other methods. Since the Wu OBA-based coarse alignment method relies entirely on sensor information, its results quickly diverge within a few seconds after obtaining a rough attitude at the beginning of motion. The sensor biases remain uncompensated throughout this method. For the Huang OBA-based coarse alignment method, the incorporation of sensor biases into the estimated state, along with the use of the dynamic OBA approach, effectively mitigates the divergence issue. Nevertheless, as the method still fully relies on satellite information, its alignment results are significantly affected when the satellite signal quality is poor, leading to suboptimal estimation performance over the entire period. In contrast to these OBA-based methods, the proposed approach employs the designed quasi-uniform quaternion generation method to estimate the initial state and then determines the most probable initial state using the maximum likelihood of the Schweppe likelihood ratio. Compared to OBA-based methods, which require valid satellite information over multiple time instants, the proposed method requires only two valid satellite observations to complete the initial estimation. Additionally, the transition from coarse to fine alignment can be determined based on the covariance of the maximum likelihood state estimate. It is also worth discussing that some initial estimates in the proposed method may lead to the divergence of the Kalman filter. However, this does not affect the final estimation results. Since diverged hypotheses exert minimal influence on the overall likelihood ratio computation, the final estimation results remain unaffected. From (13), assume that hypothesis $H_{j}$, leading to $P_{\nu_{k}\nu_{k},j}\gg P_{\nu_{k}\nu_{k},i}$. This causes a significant decrease in the likelihood ratio $\lambda_{ij}$, indicating that $H_{i}$ is more reliable. Conversely, if $H_{i}$ diverges, the likelihood ratio increases, suggesting that $H_{j}$ is more reliable. Thus, the divergence of an initial hypothesis does not affect the final result of the maximum likelihood estimation.

## 6. Conclusions

In this article, aiming at the in-motion initial alignment problem, the proposed method utilizes the proposed quasi-uniform quaternion generation method to estimate the unknown state. Then, the maximum likelihood is used to select the most probable hypothesis by comparing the Schweppe likelihood ratios between any two hypothesis distributions derived from the prediction of Kalman. The experiment results demonstrate the proposed method is capable of accurate estimation even with poor satellite signal quality and low-cost MEMS-IMU. For low-quality INS, a small number of initial estimates is sufficient to achieve excellent alignment results. However, the challenge of balancing the number of initial hypotheses, computational time, and real-time performance requires further investigation in future research.

## Figures and Tables

**Table 1:** Sensors Parameters.

Experiment Equipments Parameters (Allan Analysis)			
MEMS-IMU	gyroscope	Bias	−250°/h∼250°/h
		Bias Instability	0.5269°/h
		Random Walk	0.1426°/h
	accelerometer	Bias	750 μg
		Bias Instability	0.068 mg
		Random Walk	0.079 m/s/h
GPS	Position White Noise		3 m
	Velocity White Noise		0.15 m/s
AHRS	gyroscope Bias Instability		0.01°/h
	Angular Random Walk		0.003°/h
	Accelerometer Bias Instability		μg
RTK	Velocity Random Walk		0.003 m/s/h
	Horizontal position accuracy		8 mm
	Vertical position accuracy		15 mm

**Table 2:** RMSE errors of various methods.

Items	Proposed Method (1300 s∼*end*)	Wu OBA (1300 s∼*end*)	Huang IMCA (1300 s∼*end*)	USQUE (1300 s∼*end*)	USQUE with OBA (1300 s∼*end*)
Pitch (°)	0.2620	7.5328	0.4194	0.2668	0.2694
Roll (°)	0.2057	6.3555	0.7417	0.2270	0.2267
Yaw (°)	22.2248	128.2715	29.7970	26.2894	24.0499
Eastern Velocity (m/s)	0.4158	\	\	0.4382	0.4311
Northern Velocity (m/s)	0.4329	\	\	0.4524	0.4448
Up Velocity (m/s)	0.0977	\	\	0.0850	0.0825
Eastern Position (m)	2.7577	\	\	2.7616	2.7622
Northern Position (m)	2.2062	\	\	2.2041	2.2046
Up Position (m)	1.1242	\	\	1.0067	1.0973

## Data Availability

The data and code presented in this study are available on https://github.com/zhongluu/CoarseAlignment.git (accessed on 28 Feburary 2025).
